# Radiological tissue equivalence of deformable silicone‐based chemical radiation dosimeters (FlexyDos3D)

**DOI:** 10.1002/acm2.12658

**Published:** 2019-06-11

**Authors:** Yi Du, Ruoxi Wang, Meijiao Wang, Haizhen Yue, Yibao Zhang, Hao Wu, Weihu Wang

**Affiliations:** ^1^ Key laboratory of Carcinogenesis and Translational Research (Ministry of Education/Beijing), Department of Radiation Oncology Peking University Cancer Hospital & Institute Beijing China

**Keywords:** FlexyDos3D, gel dosimetry, silicone dosimeter, tissue equivalence

## Abstract

FlexyDos3D, a silicone‐based chemical radiation dosimeter, has great potential to serve as a three‐dimensional (3D) deformable dosimetric tool to verify complex dose distributions delivered by modern radiotherapy techniques. To facilitate its clinical application, its radiological tissue needs to be clarified. In this study we investigated its tissue‐equivalence in comparison with water and Solid Water (RMI457). We found that its effective and mean atomic numbers were 40% and 20% higher and the total interaction probabilities for kV x‐ray photons were larger than those of water respectively. To assess the influence of its over‐response to kV photons, its HU value was measured by kV computed tomography (CT) and was found higher than all the soft‐tissue substitutes. When applied for dose calculation without correction, this effect led to an 8% overestimation in electron density via HU‐value mapping and 0.65% underestimation in target dose. Furthermore, depth dose curves (PDDs) and off‐axis ratios (profiles) at various beam conditions as well as the dose distribution of a full‐arc VMAT plan in FlexyDos3D and reference materials were simulated by Monte Carlo, where the results showed great agreement. As indicated, FlexyDos3D exhibits excellent radiological water‐equivalence for clinical MV x‐ray dosimetry, while its nonwater‐equivalent effect for low energy x‐ray dosimetry requires necessary correction. The key findings of this study provide pertinent reference for further FlexyDos3D characterization research.

## 1 INTRODUCTION

With the advances of modern radiation treatment techniques featured by intensity modulated radiation therapy (IMRT) and volumetric modulated arc therapy (VMAT) in clinic, three‐dimensional (3D) radiation dosimetry has gained significant research efforts to keep pace with radiation delivery development to safeguard cancer patients that need to receive radiotherapy treatment. Since Gore et al.^1^ proposed gel dosimeters for radiation dose distribution measurement, increasing research interest has been directed to the field of gel dosimetry. Currently, as the only *true 3D dosimetric tool*,^2^ gel dosimeters possess substantial potential in performing high‐resolution 3D integral dose measurement.^3^, ^4^ Gel dosimeters have been successfully applied in highly complex radiation measurement issues such as small field dosimetry^5^, ^6^ and MRI‐linac dosimetry,^7^, ^8^, ^9^ where 3D dose measurement is totally challenging for conventional dosimetric tools.

Over the past decade, two novel gel‐type dosimeters have been proposed, both of which work in similar physical principles as radiochromic gel dosimeters but are not of hydrogel matrix: the polyurethane dosimeter known as Presage and the silicone dosimeter as FlexyDos3D. Proposed in 2003^10^ and driven by sustained scientific efforts^11^ on modification, characterization, and application, Presage has become a commercial product available for radiotherapy community (Heuris Inc, Lawrence, NJ, USA). FlexyDos3D, first proposed in 2015,^12^, ^13^, ^14^ is relatively young and still at the early stage of research and development.

FlexyDos3D is made of silicone elastomer with radiochromic leuco dyes and halogens as radiosensitive agents. When exposed to ionizing radiation, the color forming leuco dyes react with free radical initiators in the halogens. The reactions subsequently induce changes in optical absorption, which are quantitatively related to the localized absorbed dose and therefore the dose distribution can be remapped via calibration. Since silicone elastomer, the matrix material, is optically transparent and mechanically flexible, FlexyDos3D is not only an ideal dosimeter to fabricate deformable anthropomorphic dosimeter phantoms^12^, ^13^ but also suitable for optical‐CT readout without artifacts caused by sample flasks or Schlieren bands.^15^ In the meantime, the chemical recipe of FlexyDos3D has been optimized by *Høye* et al.^16^ to exhibit desired dose‐rate independent and linear dose response.

From the perspective of clinical radiotherapy quality assurance (QA), an ideal dosimeter should be radiologically tissue‐equivalent over the energy range of radiation beams. The water‐equivalence of hydrogel and Presage has been evaluated with valuable references for research and clinical practice.^17^, ^18^, ^19^ While De Deene et al.^12^ has reported that FlexyDos3D dosimeters of the original chemical formula have larger mass attenuation coefficients for low energy, its tissue equivalence has not been fully studied yet. In this paper, we investigate the tissue equivalence of the optimized dose‐rate independent FlexyDos3D in a wellrounded hybrid approach by theoretical calculation, x‐ray CT measurement and Monte Carlo simulation. The results are compared with water as benchmark and Solid Water (RMI457) as reference, a commercial water substitute for radiotherapy dosimetry.^20^

The rest of the paper is organized as follows: Section 22 details the elemental composition of FlexyDos3D and the methods to assess its tissue‐equivalence; Sections 33 and 44 presents and discusses the radiological properties and dosimetric quantities of interest; Section 55 summarizes the major conclusions.

## 2 MATERIALS AND METHODS

The fabrication method of the finely tuned dose rate independent FlexyDos3D was first presented. Then its elemental composition was derived and used as material information for the following theoretical calculation and Monte Carlo simulation. In this work, tissue‐equivalence were assessed from four aspects: (a) key physical parameters of radiological interest were first calculated, including equivalent electron density, effective atomic number, and interaction probabilities; (b) Housefield unit (HU) value was measured by kilovoltage (kV) computed tomography (CT) and the impact of its deviation from water to treatment planning system (TPS) dose calculation were evaluated; (c) magavoltage (MV) relative dosimetry indices, that is, percentage depth doses (PDD) and profiles were modeled by Monte Carlo simulation; (4) a toy volumetric modulated arc therapy (VMAT) treatment was computationally delivered to FlexyDos3D and reference materials by Monte Carlo and their dose distributions were compared.

### 2.1 FlexyDos3D fabrication and elemental composition

To fabricate dose‐rate independent FlexyDos3D dosimeters,^16^ three chemical products are required: (a) silicone elastomer kit (Sylgard 184, Dow Corning Corporation), which provids elastomer base and curing agent (CA) separately; (b) chloroform (Sigma‐Aldrich); (c) leuco‐malachite green dye (Sigma‐Aldrich). The Sylgard 184 base and CA are mixed by 10:1 (w/w), and then 1%(w/w) chloroform with 0.26% (w/w) dissolved leuco‐malachite green (LMG) is added to the mixture. After thorough stirring, air bubbles are outgassed under vacuum condition. The bubble‐free mixture is then poured into designed moulder, which are then left in dark room to cure for 48 h.

Based on the chemical formula and weight fraction of each component, the equivalent elemental composition of FlexyDos3D was determined as listed in Table 1 with water and Solid Water as reference.

**Table 1:** Elemental composition and corresponding fractional weight for materials of interest.

Material	Fractional weight (%, *w/w*)						
	H	C	N	O	Si	Cl	Ca
Water^a^	11.19	\	\	88.81	\	\	\
Solid Water (RMI457)^a^	8.09	67.22	2.40	19.84	\	0.13	2.32
FlexyDos3D	8.24	33.01	0.0007	21.08	37.62	0.05	\

a From ICRU report 44: tissue substitutes in radiation dosimetry and measurement.

### 2.2 Electron density, effective and mean atomic number calculation

Electron density, effective atomic number and mean atomic number are key theoretical quantities to radiological evaluate tissue‐equivalence. For each material, the electron number per cubic cm (*ρ_e_*), electron number per gram (*n_e_*) and relative electron density (*r_e_*) (named as *real density* in *Eclipse* (Varian Medical Systems Inc.)) were calculated by:

(1) $$\rho_{e}=N_{A}\cdot \rho \cdot \sum_{i=1}^{n}w_{i}\cdot \left(\frac{Z_{i}}{A_{i}}\right),$$

(2) $$n_{e}=\frac{\rho_{e}}{\rho },$$

(3) $$r_{e}=\frac{\rho_{e}}{\rho_{e,water}},$$

where *N_A_* is the Avogadro's number, *ρ* is the mass density, and *Z_i_*, *A_i_,* and *w*_i_ are respectively the atomic number, atomic mass number and weight fraction of element *i*.

According to ICRU Report No. 35,^21^ the mean atomic number Z_mean_ was calculated by eq. (4). In the meantime, the effective atomic number *Z*_eff_ was determined using the classic Mayneord formula^22^, ^23^ as in eq. (5):

(4) $$Z_{\text{mean}}=\sum_{i=1}^{n}\left(f_{i}\cdot \frac{Z_{i}^{2}}{M_{A_{i}}}\right)/\sum_{i=1}^{n}\left(f_{i}\cdot \frac{Z_{i}}{M_{A_{i}}}\right),$$

(5) $$Z_{\mathrm{eff}}=\sqrt[{2.94}]{\sum_{i=1}^{n}a_{i}Z_{i}^{2.94}},$$

where *f_i_* is the mass fraction, *a_i_* is the electron fraction, and *M_Ai_* is the molar mass of element *i*.

### 2.3 Photon interaction probabilities and electron stopping powers

For kV imaging and MV treatment photon beams, photoelectric absorption, Compton scattering, and pair production effect are governing interactions between x‐ray photons and the materials that photons traverse, and the interaction probabilities can be defined by mass attenuation coefficients as:

(6) $$\left(\mu /\rho \right)=\left(\tau /\rho \right)+\left(\sigma /\rho \right)+\left(\kappa /\rho \right),$$

where (τ/ρ), (σ/ρ), (κ/ρ) are the mass attenuation coefficient of photoelectric absorption, Compton scattering, and pair production, respectively, and (μ/ρ) is the total mass attenuation coefficient. The mass attenuation coefficients of different materials were calculated using the NIST XCOM database with *mixure rule* option over the energy span from 1 to 20 MeV.^24^

For electron radiotherapy beams, projectile electrons from a linac deposit energy along traverse paths in travelling media by exciting and ionizing atoms. In the process of interaction with electrons, stopping powers are recommended quantities to evaluate tissue equivalence in ICRU Report 44.^25^ Herein, we used NIST ESTAR database^26^ with the *mixture rule* option to calculate the mass collisional stopping powers (S_c_ */ρ*), mass radiative stopping powers (*S_r_/ρ*), and total mass stopping powers (S_t_/*ρ*) of different materials over the energy range from 10 keV to 20 MeV:

(7) $$\left(S_{t}/\rho \right)=\left(S_{c}/\rho \right)+\left(S_{r}/\rho \right).$$

### 2.4 KV x‐ray CT based HU value and the derived electron density

**Figure 1:**
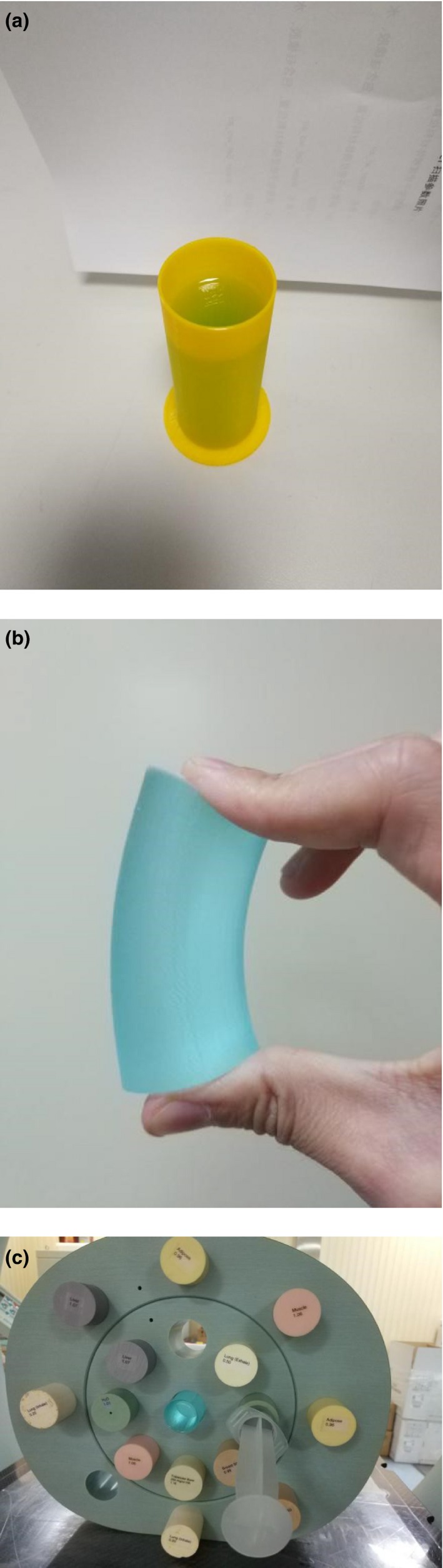
(a) and (b) Fabricated FlexyDos3D sample insert; (c) CIRS phantom with the FlexyDos3D in center. Note that the syringe housed by a plastic‐water ring contained deionized water

In radiotherapy treatment planning, dose calculation is based on the equivalent patient/phantom electron density distribution derived from kV CT images via HU value to electron density calibration. Herein, we first measured the HU value of FlexyDos3D and compared with that tissue substitutes. Following the fabrication procedure in Section 2.2.1A, a cylindrical FlexyDos3D insert phantom (diameter = 30 cm, height = 8 cm) was made as shown in Figs. 1 and 1(b), which was suitable for our electron density phantom (Model 062M, CIRS). The CIRS phantom was scanned on a CT‐Sim (SOMATOM Open, Siemens) using pelvis imaging protocol (Voltage = 120 kV, Effective mAs = 110, pitch = 0.8, SliceThickness = 2 mm). During CT scanning, the FlexyDos3D inserted was positioned at the center of the phantom while several other vendor‐provided tissue‐substitute inserts were put in certain holes as shown in Fig. 1(c). After scanning, the CT Digital Imaging and Communications in Medicine (DICOM) images were imported into the clinical treatment planning system Eclipse (version 13.6), and the inserts of interest were contoured as individual structures. The mean HU value and corresponding standard variation of each contoured structure were calculated.

The derived electron density of FlexyDos3D by HU‐value to electron‐density conversion was calculated. Note that the conversion curve, also used for clinic, was measured with the same electron density phantom and modeled by Eclipse. As shown in Section 3.D3.4, we found that the electron density of FlexyDos3D was over‐estimated via direct HU‐value conversion. To assess the impact of HU value deviation from water on dose calculation in treatment planning, we made a simple four‐field static conformal treatment plan (classic box‐field plan) targeting the contoured central FlexyDos3D insert with 6‐MV photon beam. The dose prescription of the target volume was set as with 2 Gy and 100% coverage. The dose distribution was recalculated with the same beam configuration while the HU value inside the target structure was reset as HU = 0 (water) and HU = 20 (which related to the correct electron density of FlexyDos3D). The dose distributions of the plans with and without HU correction were compared.

### 2.5 MV photon depth doses and profiles by Monte Carlo simulation

Percentage depth dose curves (PDDs) and off‐axis ratios (lateral profiles) of MV x‐ray photons in water are imperative dosimetric data describing beam models for radiotherapy dose calculation. In order assess water‐equivalence of FlexyDos3D, Monte Carlo simulations were carried out to obtain PDDs and profiles in aforementioned three different materials. The corresponding curves in different materials ware inter‐compared to evaluate possible energy over‐response of FlexyDos3D.

**Figure 2:**
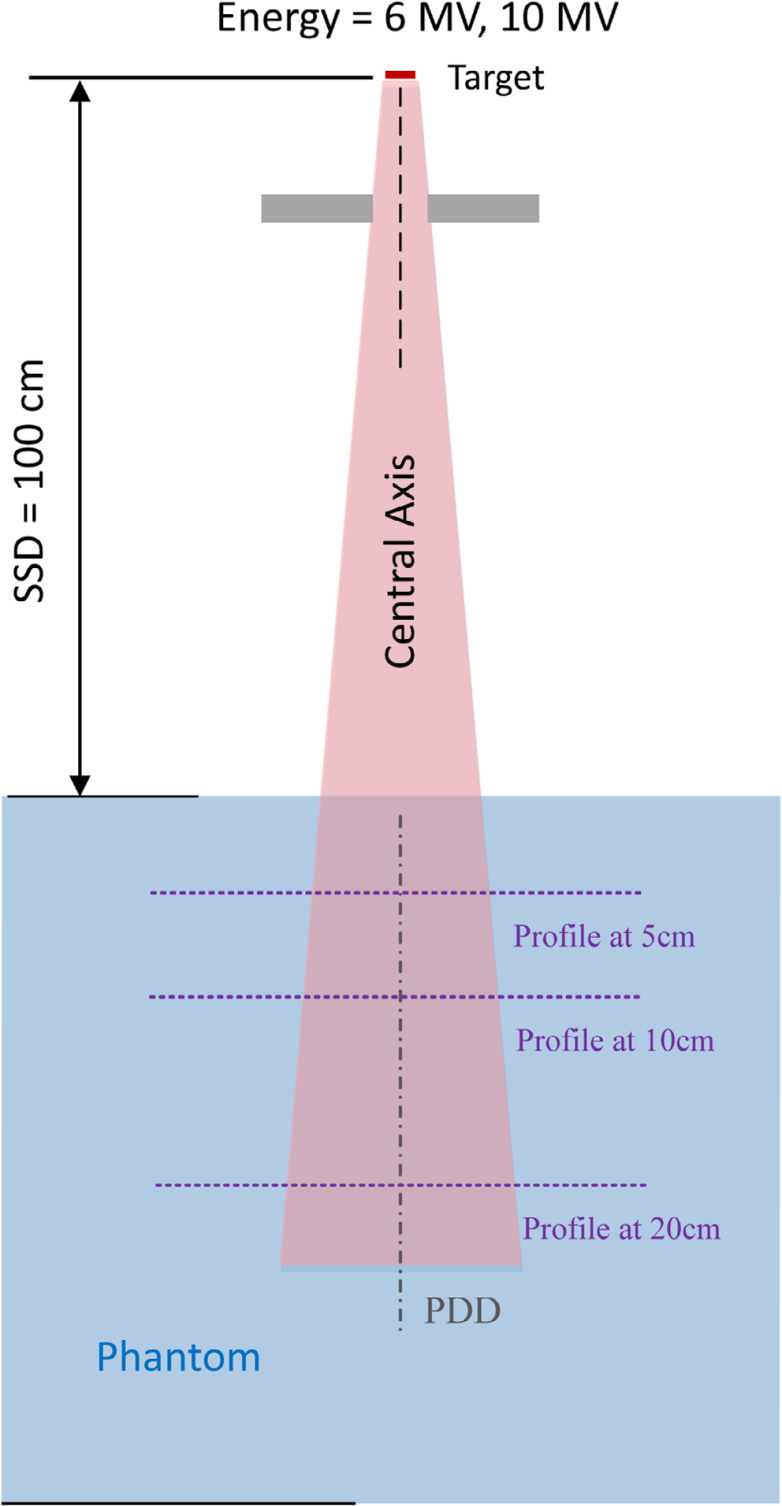
Schematic of the Monte Carlo simulation scenario for PDD and profile calculation

The simulation geometry was setup as in Fig. 2 with a bulk phantom as large as 50 cm × 50 cm × 50 cm. The source‐to‐surface‐distance (SSD) was set at 100 cm. The nominal beam energy was 6 and 10 MV, which are the most frequently used in clinic. Three field‐size conditions were calculated, respectively: a typical small field as 2 cm × 2 cm, the reference field as 10 cm × 10 cm, and a very large field as 40 cm × 40 cm.

PDD and profiles were scored in the voxelized phantom while the material in the phantom was homogeneously defined as FlexyDos3D, Solid Water (RMI457), and water, respectively, with the voxel size as 0.2 cm × 0.2 cm × 0.2 cm. The scoring depth of PDD was set from 0.1 to 30.1 cm, while the range of profiles varies according to the field sizes.

All the simulations were performed with BEAMnrc/DOSXYZnrc (version 2017, National Research Council of Canada), hosted on an Linux cluster.^27^ Manufacturer‐distributed phase space files scored above the upper jaw were used to sample initial particle state, whereas the jaws and collimators are modeled according to geometry description provided by the manufacturer.^28^ Particle histories were set according to the field size, ranging from 4 × 10^9^ to 5 × 10^10^, in order to achieve a mean statistical uncertainty of 0.5% (*k* = 1) over all the voxels with doses greater than 50% of the maximum dose. The cross‐section data were generated by the PEGS4 program^27^, ^29^ using composition and density information in Tables 1 and 2. The photon and electron cutoff energy were set as 0.01 and 0.7 MeV respectively. The electron range rejection was set to 1 MeV inside the scoring phantom, while no other variance reduction techniques were employed in the simulation.

**Table 2:** Electron density, effective atomic number, and mean atomic number for materials of interest.

Material	*ρ*	*ρ_e_*	*n_e_*	*r_e_*	*Z_eff_*	*Z_mean_*
	(g/cm^3^)	(10^23^/cm^3^)	(10^23^/g)			
Water	1.00	3.3416	3.3416	1.0000	7.4166	6.6000
Solid water (RMI457)	1.03	3.3469	3.2494	1.0016	7.3969	5.9600
FlexyDos3D	1.03	3.3481	3.2506	1.0019	10.3474	8.4165

Alongside the simulation results, detector‐measured PDD and profile curves were also given. The reference‐field and large‐field measurements were from TrueBeam Representative Beam Data (RBD).^30^, ^31^ Since the 2 cm × 2 cm small‐field data were not provided in RDB, we used the measured data on a TrueBeam machine with PTW 60016 Diode‐P detector mounted on PTW BEAMSCAN water tank.

### 2.6 Toy VMAT treatment delivery by Monte Carlo simulation

To estimate the tissue equivalence of FlexyDos3D in a more realistic scenario, that is, gel dosimeter based end‐to‐end dose verification,^32^ where the beam configuration and dose distribution are much more complex than static field irradiation, we made a toy VMAT treatment plan and then computationally performed the delivery onto a phantom by Monte Carlo simulation. The phantom herein was an MRI‐linac dynamic phantom (Model 008M, CIRS), inside which there was a movable cylindrical rod. We scanned the phantom on our CT‐Sim with an isotropical resolution of 1.25 mm and transferred its CT images to Eclipse for treatment planning.

**Figure 3:**
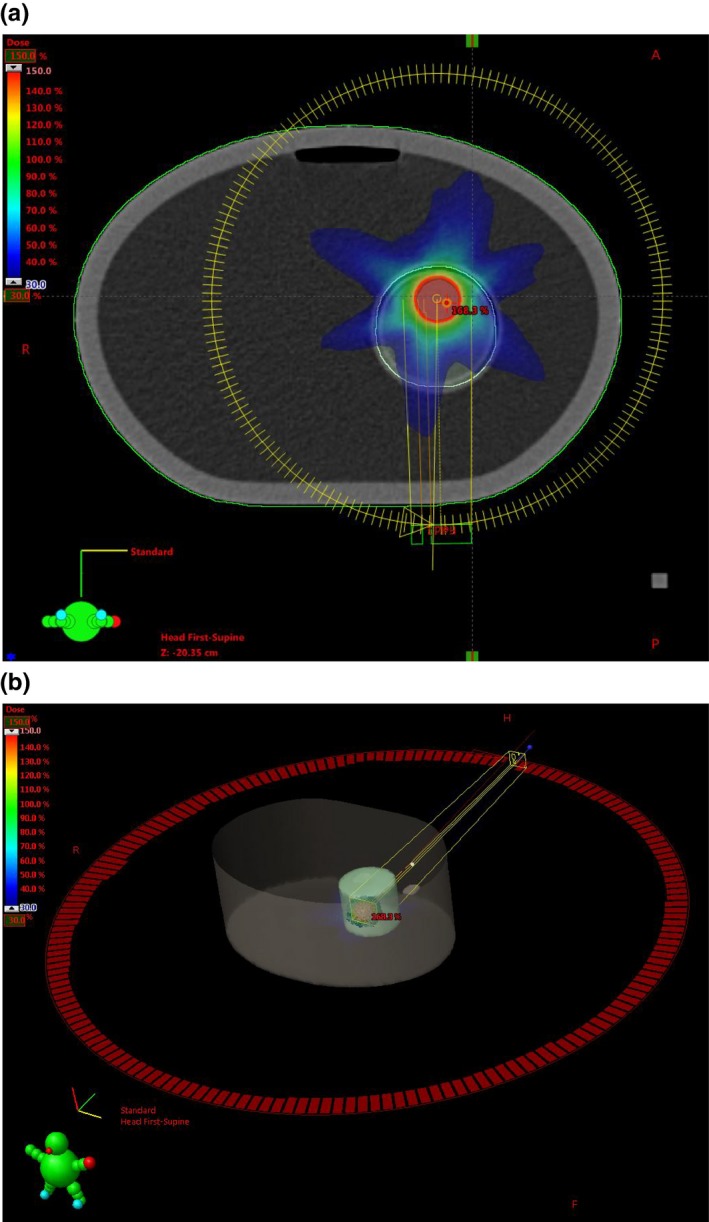
(a) Slice view of the dose distribution in the dose‐maximum plane where the gross tumor volume contour could be easily identified. (b) BEV of the volumetric modulated arc therapy (VMAT) treatment plane with BODY and DOS structures shown.

We contoured part of the rod as a structure (denoted as DOS) to mimic a volumetric dosimeter inserted into the phantom. Inside DOS, we contoured a tumor‐like small volume as toy gross tumor volume (GTV), which was targeted by a one‐course full‐arc VMAT plan as shown in Fig. 3. After treatment planning in Eclipse, the treatment plan and structure information (stored in DICOM‐RP and DICOM‐RT files) were imported into our Monte Carlo platform as in Section 2.E2.6 for further processing.

With consideration of computing resource limits, the resolution of the phantom was down‐sampled to 3 mm to achieve converged dose scores within reasonable time. An in‐house python script was used to assign water as the material of the phantom except DOS. Similarly, the material inside DOS was assigned to water, solid water, and FlexyDos3D interchangeably to obtain different dose distribution within corresponding material. In DOSXYZnrc, *Source 21* was used to simulate the VMAT beam delivery, where a BEAMnrc program was compiled as a particle source (dynamic library) for the DOSXYZnrc simulation.^33^ The movement of jaws and MLCs, the rotation of the collimator, and the rotation of the gantry within the frame of reference of the irradiated phantom were all synchronized with MU indices. Primary particle histories were set at 1 × 10^10^ such that a mean statistical uncertainty of 0.5% (*k* = 1) over the voxels with at least 50% global maximum dose.

## 3 RESULTS

### 3.1 Electron density, effective, and mean atomic number

The calculated electron density, effective atomic number, and mean atomic number values of water, Solid Water, and FlexyDos3D are listed in Table 2. The density of FlexyDos3D, equal to Solid Water as 1.03 g/cm^3^, is 3% higher than water. The electron density, *ρ_e_*, of FlexyDos3D is about 0.2% higher than water, while the electron number per gram, *n_e_*, is about 2.8% smaller than water. The calculated Z_eff_ and *Z*_mean _values of FlexyDos3D are approximately 40% and 20% higher than water, respectively, which can be attributed to its high silicon composition (37.62% w/w).

### 3.2 Photon interaction probabilities

**Figure 4:**
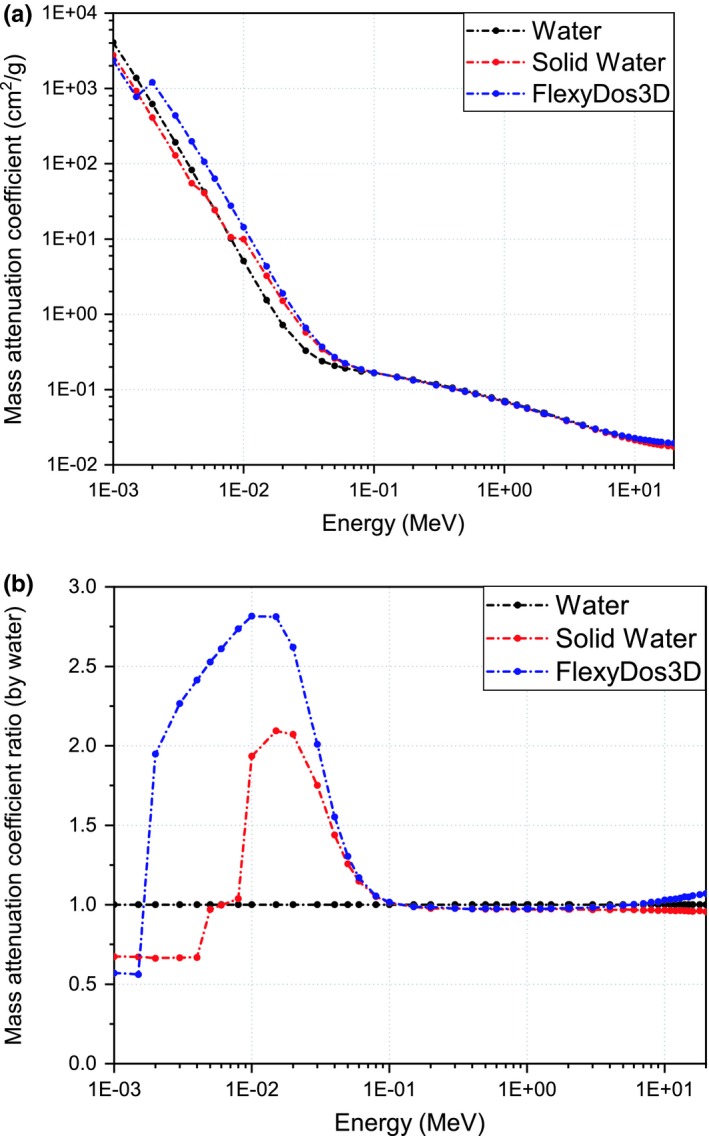
Interaction probabilities between materials and photons in the energy range of 1 keV to 20 MeV: (a) total mass attenuation coefficients, (b) mass attenuation ratio normalized by water.

The total mass attenuation coefficients (μ/ρ) of FlexyDos3D, Solid Water, and water are plotted in Fig. 4(a), and the relative ratios normalized by water are shown in Fig. 4(b). According to the general trend, the curves can be separated into three parts: in the energy range below 100 keV, the characteristic K‐edge of FlexyDos3D can be easily identified with the peak ratio value around 10 keV, and the attenuation coefficients are generally larger than both water and Solid Water, while the discrepancy comes negligible as the photon energy increases to 100 keV; in the range between 100 keV and 1 MeV, (μ/ρ) of FlexyDos3D, Solid Water and water are almost the same to each other with difference less than 2%; for the energy beyond 1 MeV, the curves become slightly divergent, where (μ/ρ) of FlexyDos3D is 5% higher than water and Solid Water exhibits 2% under‐response to water.

**Figure 5:**
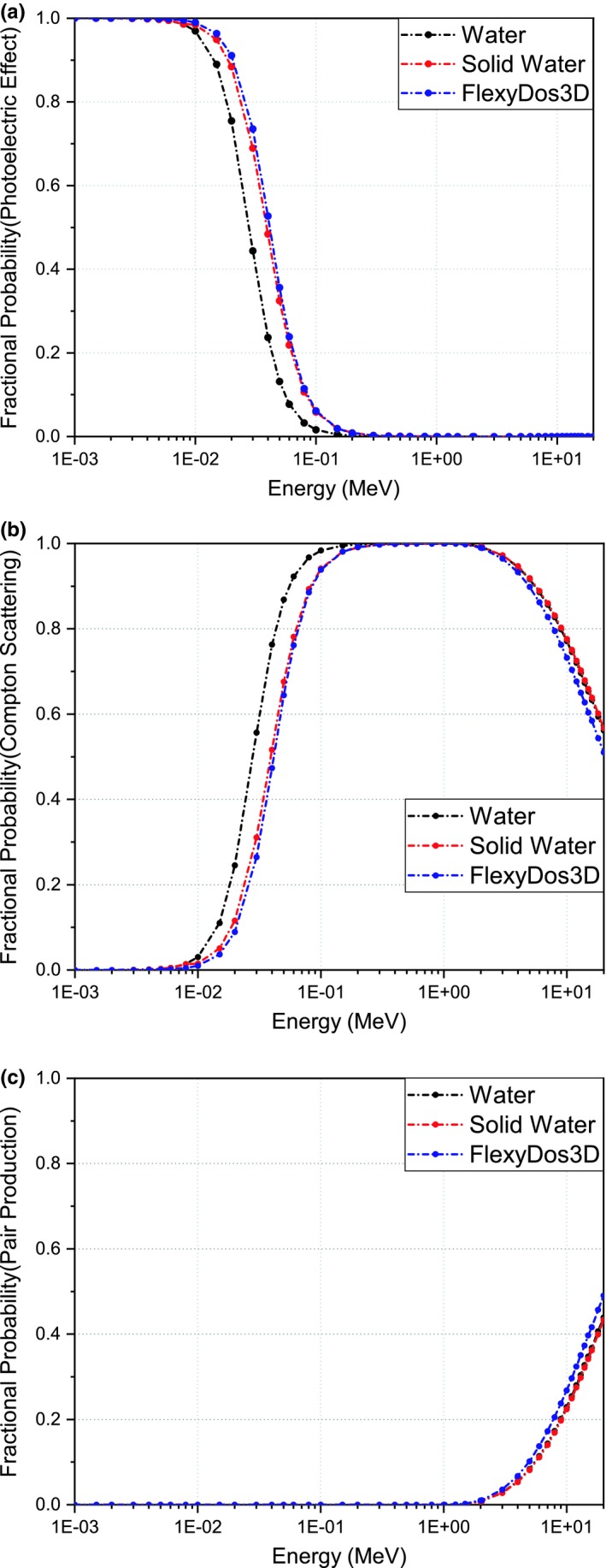
Fractional interaction probabilities of (a) photoelectric effect, (b) Compton scattering, and (b) pair production effect in the energy range over 1 keV to 20 MeV.

The fractional interaction probabilities of photoelectric effect (τ/μ), Compton scattering (σ/μ) and pair production effect (κ/μ) are plotted in Figs. 5(a)–5(c). It is obvious that photoelectric effect plays a dominant role for all the three materials when photon energies are below 100 keV. Since the cross‐section of photoelectric effect is approximately proportional to cube of atomic number,^23^ that is *Z^3^*, the difference of fractional probabilities around 50 keV between FlexyDos3D and water is as large as 20%. When photon energies increase all the way to 20 MeV, Compton scattering becomes dominant, the cross‐section of which is closely related to electron density. As calculated in Section 3.3.1A, *r_e_* between FlexyDos3D and water is as small as 0.19%. This can well explain the negligible probability difference. For photons with energies beyond the threshold value of 1.02 MeV, pair production starts to occur and the interaction probability increases slightly as photon energy goes up. Since the cross‐section of pair production is dependent on *Z^2^/A*, the differences between FlexyDos3D Solid Water and water indicate that FlexyDos3D has a slightly larger *Z^2^/A* value than both Solid Water and water.

### 3.3 Electron stopping powers

**Figure 6:**
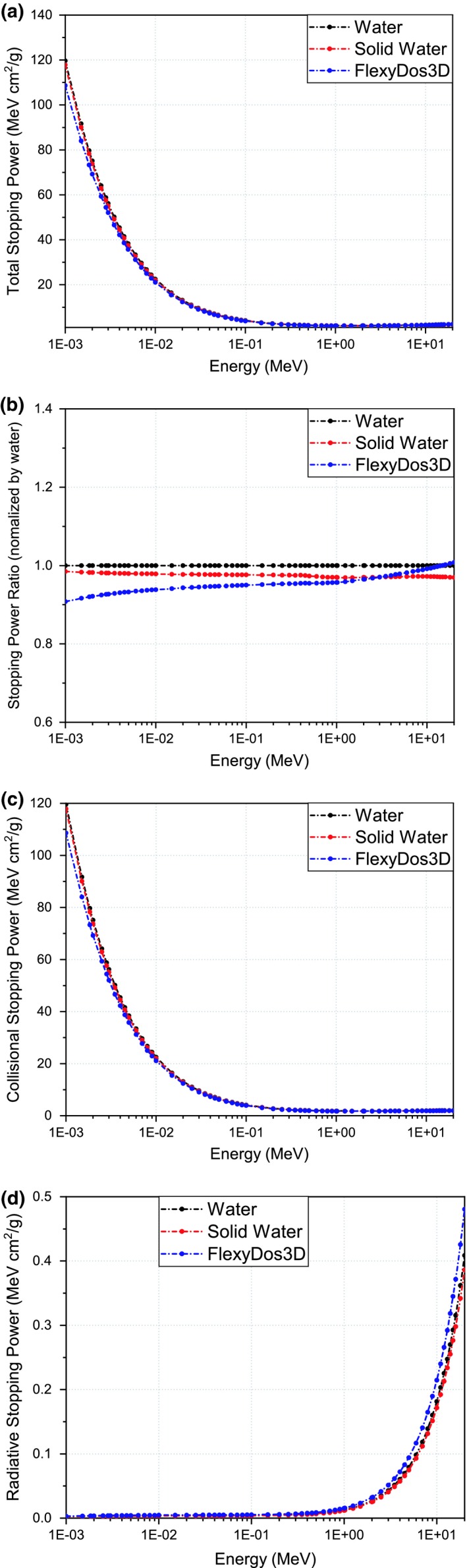
Stopping power plots between materials and electrons in the energy range over 1 keV to 20 MeV: (a) total stopping powers, (b) stopping power relative ratios normalized by water, (c) collisional stopping powers, and (d) radiative stopping powers.

The stopping powers of FlexyDos3D, Solid Water, and water are plotted in Figs. 6(a)6(d). We can see in Fig. 6(b) that for FlexyDos3D the difference in total stopping power with water is about 10% at 1 keV and decreases gradually to almost zero at 10 MeV and 0.5% at 20 MeV, while the difference of Solid Water with water increases from around 1.5% at 1 keV to about 5% at 20 MeV. As indicated in Figs. 6(a), 6(c) and 6(d), collisional stopping power dominates for all three materials over the energy range, which accounts for almost 100% of total stopping power at 1 keV and about 80% at 20 MeV. Compared with water, FlexyDos3D exhibits about 10% lower stopping power over the energy from 1 keV to 10 MeV, but the discrepancy gets narrower gradually to less than 1% from 10 to 20 MeV.

### 3.4 HU value measured by kV x‐ray CT

**Figure 7:**
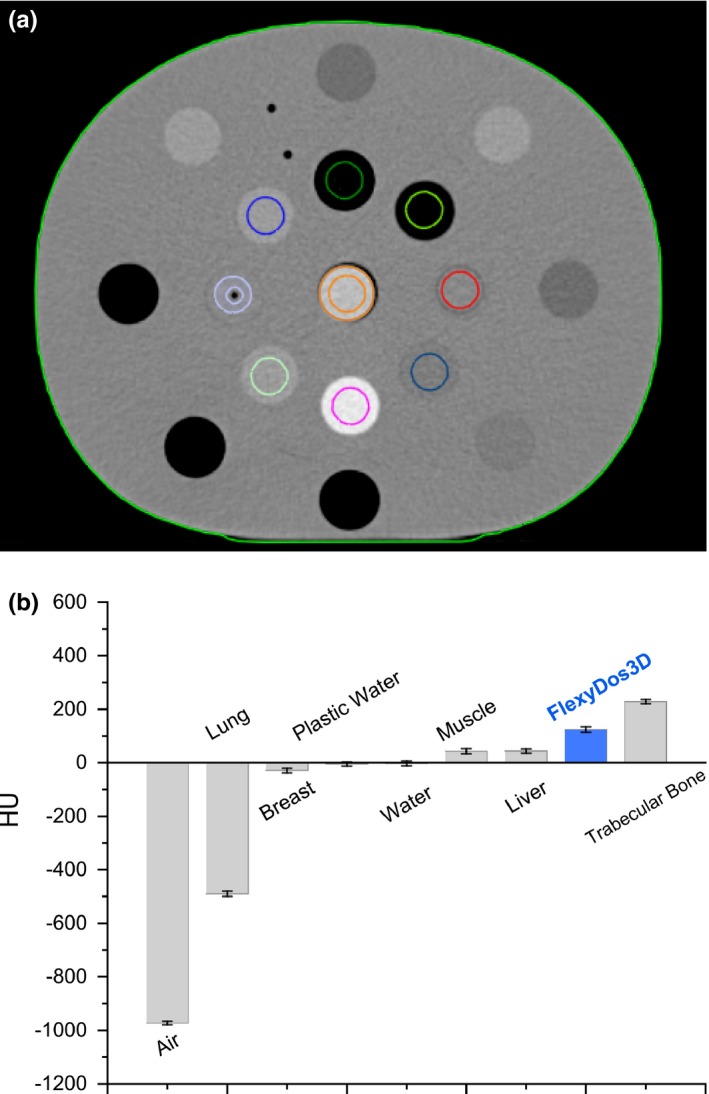
HU values of FlexyDos3D and tissue substitute inserts inside the CIRS phantom: (a) computed tomography (CT) image with contoured ROIs, (b) HU values of different materials with error bars.

The HU values of tissue substitute rods were calculated within circular ROIs as shown in Fig. 7(a), and the results are listed in Fig. 7(b). The uncertainties drawn as error bars are expressed as the standard deviation (*k* = 1) of HU values in each contoured structure. It is evident that for kV x‐ray beam FlexyDos3D has a high HU value (123.9 ± 9.5), which is much larger than water (−2.79 ± 8.7) and other soft tissue substitutes (for example, HU_Liver_ = 43.6 ± 7.3 and HU_Muscle_ = 43.2 ± 9.2) and 53.9% of the trabecular bone substitute (228.255 ± 8.5). Although silicone has a similar density to water, HU values from CT scans reveal FlexyDos3D performs more like high‐density tissues rather than soft tissues. This can be attributed to the fact in Section 3.B that FlexyDos3D has larger photon interaction probabilities than water for photons with energies less than 100 keV, which are the major x‐ray photons produced by x‐ray tubes and utilized in clinic for medical imaging purpose.

### 3.5 Electron density overestimation effect on dose calculation

**Figure 8:**
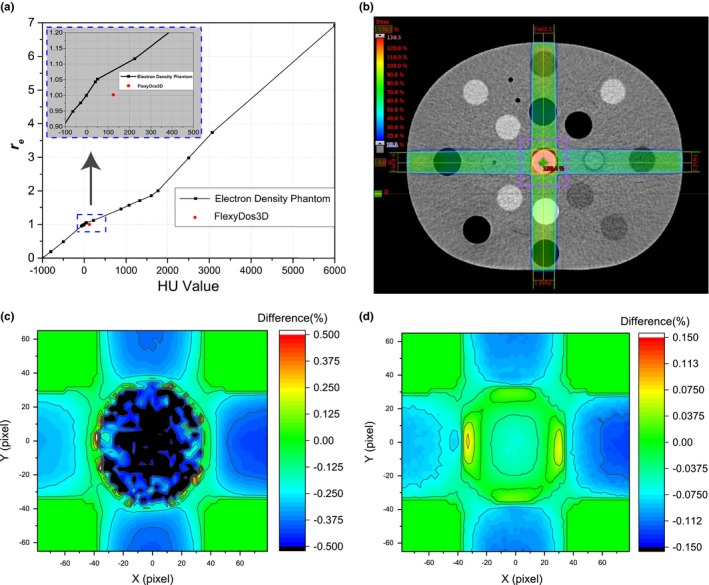
(a) HU‐value to re conversion curve in Eclipse with the FlexyDos3D data point highlighted; (b) box‐field treatment plan targeting the central structure with a rectangle ROI for dose difference analysis in (c) and (d); (c) relative dose difference between plans with HU = 124 (measured FlexyDose3D HU) and HU = 0 (water), (d) relative dose difference between planes with HU = 20 (corrected by FlexyDos3D theoretical electron density value) and HU = 0 (water). Note that the colorbar ranges in (c), and (d) are different.

The HU‐value to relative‐electron‐density (*r_e_*) conversion curve in our Eclipse is shown in Fig. 8(a), where the measured HU‐value and theoretically calculated *r_e_* pair, that is, (123.9, 1.0019) is highlighted as red dot. We can see that the real *r_e_* value of FlexyDos3D is below the conversion curve and its corresponding HU is about 20, which means that the *r_e_* derived by HU‐value conversion is larger than its real value. If uncorrected, this would lead to about 8% overestimation of *r_e_*.

To quantitatively analyze this *r_e_* over‐estimation effect on dose calculation, we calculated the 3D gamma of the dose distribution with and without HU correction. The criteria we used is 1%/1mm, and the reference was the dose distribution calculated with water replacement (HU = 0). The 3D gamma is 100% for both cases.

In the meantime, we defined relative dose difference (denoted as DIFF) as below in (7) to further evaluate the impact of electron density effect and the effectiveness of HU correction.

(7) $$\text{DIFF}\left(\%\right)=\frac{\text{Plan}(\text{HU}=x)-\text{Plan}(\text{HU}=0)}{\text{Plan}(\text{HU}=0)}\times 100\%$$

where *x* = 20 represents the dose distribution where the ROI's HU value related to the correct FlexyDos3D electron density value, and *x* = 124 represents the dose distribution where the ROI's HU value was directly measured from the x‐ray CT images.

The results in the dose‐maximum plane are illustrated in Figs. 8(c) and 8(d). We can see that without HU correction the DIFF values of the ROI are generally about 0.65% under‐estimated, ranging from −1.002% to 0.743%; those with HU correction are much smaller between −0.128% and 0.082%.

### 3.6 PDD curves

**Figure 9:**
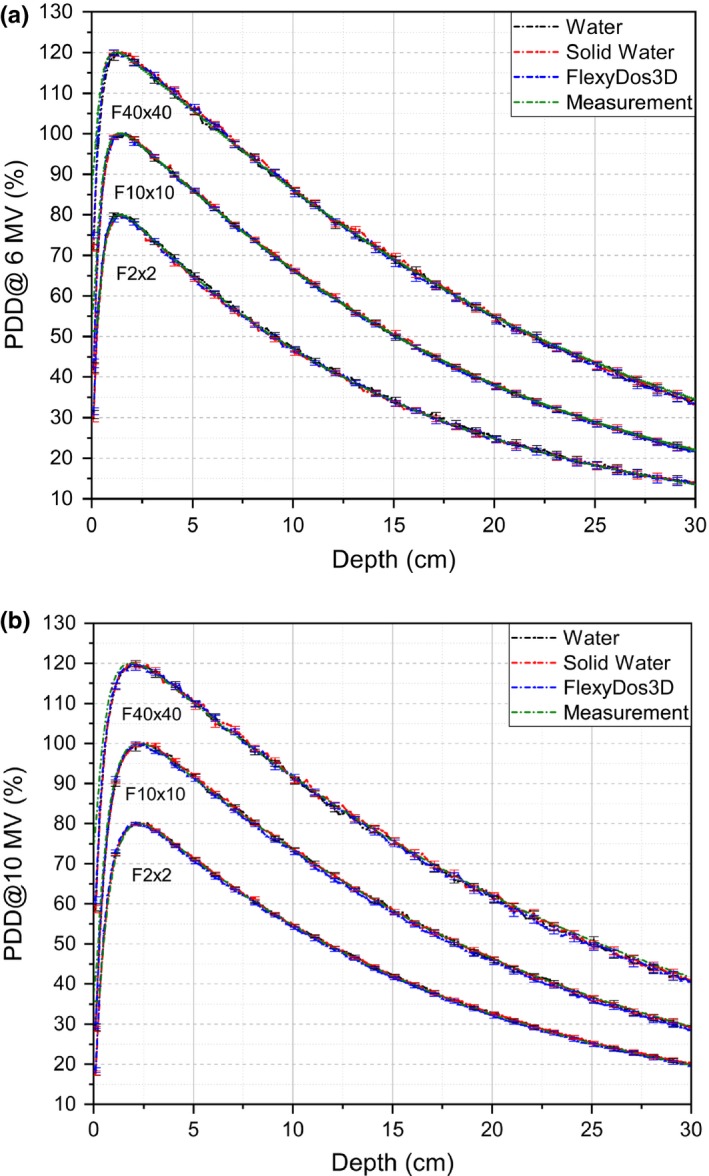
Percentage depth dose curves by Monte Carlo simulation in different materials for (a) 6‐MV photon and (b) 10‐MV photon beams for the 2 cm × 2 cm small field (weighted by 0.8), the 10 cm × 10 cm reference field and 40 cm × 40 cm large field (weighted by 1.2).

The simulated PDD curves in FlexyDos3D, Solid Water, and water at various field sizes are plotted in Fig. 9(a) for 6‐MV and Fig. 9(b) for 10‐MV. The uncertainties represented as error bars are generally less than 0.6% for 2 cm × 2 cm and 10 cm × 10 cm, and smaller than 1% for 40 cm × 40 cm.

As validation of the Monte Carlo modeling, we can see that the simulated PDDs in water agree quite well with the measurements for both small and reference fields, where the differences are blended within error bars. For the 40 cm × 40 cm field, slight differences exist in the superficial region with higher measurement values. This can be explained by the electron contamination effect on the detector.

In Fig. 9 we can see that the simulated PDDs in FlexyDos3D, Solid Water, and water change highly in phase with each: the depth dose first increases from the surface and then decreases gradually with maximum at 1.5 cm for the small and reference fields and 1.4 cm for the large field. What's more, the curves are so close to each other that the discrepancies of each point data at the same depth are blended within error bars.

### 3.7 Profiles

**Figure 10:**
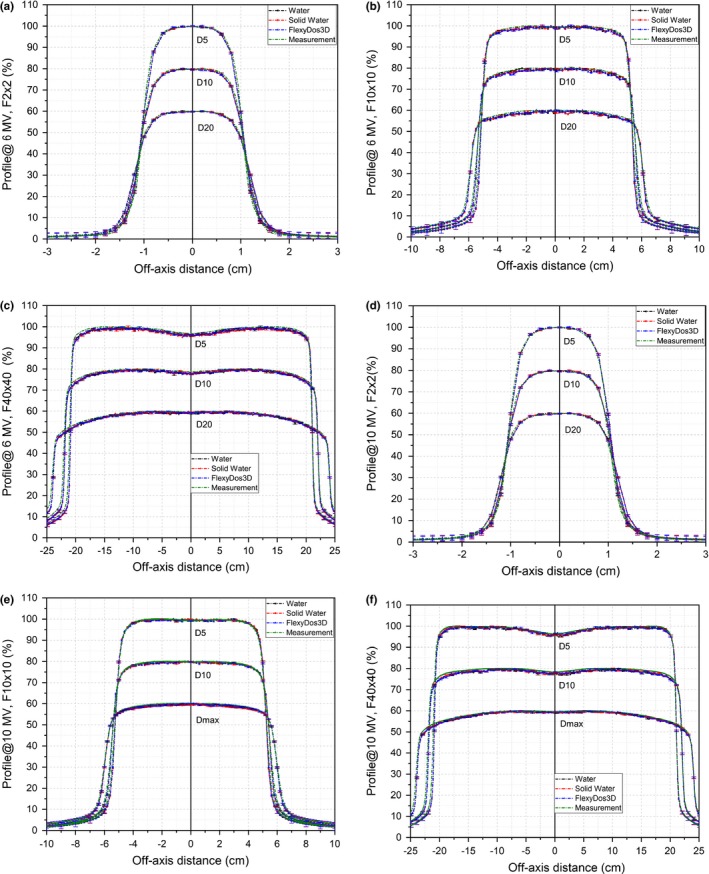
Percentage profiles by Monte Carlo simulation in different materials at various conditions for (a)–(c) 6‐MV photon and (d)–(f) 10‐MV photon beams. D5, D10, and D 20 represent data at depth = 5, 10, and 20 cm respectively.

The simulated profiles in FlexyDos3D, Solid Water, and water at various field sizes are plotted in Figs. 10(a)10(c) for 6‐MV and in Figs. 10(d)10(f) for 10‐MV. The uncertainties shown as error bars are about 0.4% for 2 cm × 2 cm and 10 cm × 10 cm in central part and become larger through penumbras to shielded areas. For 40 cm × 40 cm, the uncertainties in the central field is 0.4% and go up to about 0.6% in the shoulder area. While small discrepancies can he identified in the penumbra parts, the simulated profiles in water generally agree well with the measurements at various depths for all the three fields. In the meantime, we can see that the differences between FlexyDos3D, Solid Water and water are very small, which are also blended within error bars.

### 3.8 Toy VMAT treatment delivery by Monte Carlo simulation

**Figure 11:**
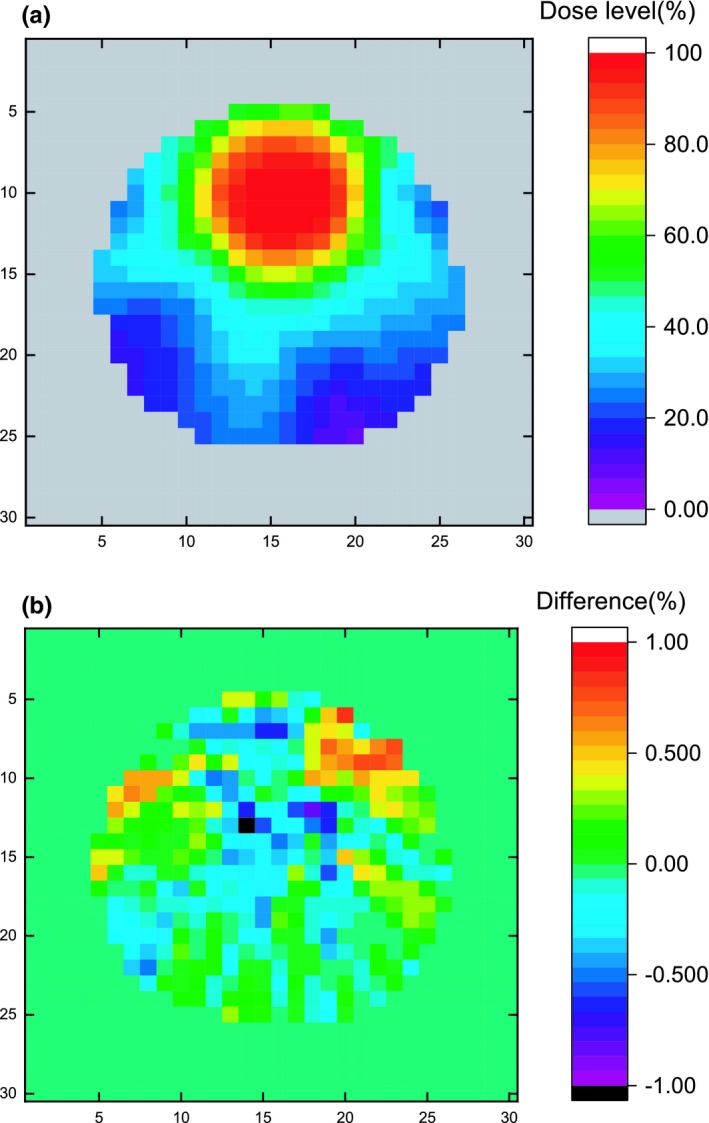
(a) Dose distribution in the dose‐maximum plane in the water‐filled DOS structure; (b) dose difference map of (a) between FlexyDos3D and water

The dose distribution in the dose‐maximum plane inside the water‐filled DOS structure is illustrated in Fig. 11(a), where we can see fast dose fall‐off around the hotspot GTV region. The relative dose difference between FlexyDos3D and water, calculated in a similar way as in eq. (7), ranges from −1.027% to 0.821%, and as shown in Fig. 11(b) the differences are generally random and we did not observe any biased effect. In the meantime, the 3D gamma with the water‐filled dose distribution as reference was calculated. The criteria we used were 3%/3 mm, 2%/2 mm, and 1%/1 mm, and the pass rates with dose interpolation were 100%, 100%, and 99.2% respectively. The high pass rates can be partly attributed to the small and unbiased dose differences as shown in Fig. 11(b), and partly to dose interpolation used in gamma calculation.

## 4 DISCUSSION

FlexyDos3D has similar mass density and electron density with water and Solid Water, which yields close Compton scattering interaction probabilities to each other. Due to the silicon composition as large as 37.6%, the effective and efficient atomic number of FlexyDos3D are much higher, which leads to higher photoelectric effect interaction probabilities. This implies that FlexyDos3D will have over‐response effect for kV photons, and therefore further correction is required for low energy x‐ray dominant dosimetry, such as kV‐based intraoperative radiotherapy and isotope‐based brachytherapy. The deviations in stopping powers between FlexyDos3D and water are less than 1% in the energy range of 10–20 MeV. While the stopping powers differ by almost 10% in kV range, based on the negligible discrepancies between the simulated PDD and profiles in different materials, we do not see any noticeable impact on dose deposition.

The HU value of FlexyDos3D measured by kV CT is larger than those soft‐tissue substitutes but smaller than the trabecular bone material, which can be attributed to its higher photoelectric interaction probabilities for low‐energy photons. Although photon beams in radiotherapy for treatment purpose are primarily in MV range, dose calculation is based on kV CT images. When using the measured HU value for dose calculation, the built‐in electron density conversion model in TPS is found to over‐estimate the electron density by about 8%. This electron density over‐estimation effect will induce underdose to the target by about 0.65%, and by HU correction the impact can be eliminated.

The simulated PDD and profiles for 6 and 10 MV validated by measurements exhibit great water‐equivalence of FlexyDos3D not only in reference fields, but also in fields as small as 2 cm × 2 cm and as large as 40 cm × 40 cm. It is noted that silicon‐based semi‐conductor detectors have been reported to overresponse in small and large fields,^34^, ^35^ but this phenomenon is not observed in FlexyDos3D, which is well worth of further investigation. As for the dose distribution comparison of the full‐arc VMAT plan computationally delivered to water and FlexyDos3D, the differences are within 1% and we do not perceive any biased error pattern.

According to previous research^36^, ^37^ and our experience, the mechanical deformability of FlexyDos3D can be tuned by modifying the elastomer base to CA ratio. This is one of its outstanding properties, which can be utilized to mimic organs of various stiffness. In this study, we only used the vendor‐recommended radio of 10:1 for tissue‐equivalence evaluation. Although changing the ratio may modify some properties of FlexyDos3D, considering the equal mass density and highly similar elemental composition between elastomer base and CA, we believe that impact to the tissue equivalence is as small as negligible and consistent conclusions still can be drawn.

## 5 CONCLUSION

FlexyDos3D, as a flexible silicone base chemical dosimeter, has the great potential to be fabricated as a deformable anthropomorphic phantom for clinical dose measurement and verification. In this study, the radiological tissue equivalence of FlexyDos3D is investigated thoroughly in: (a) theoretical parameters, (b) measured HU value, (c) simulated PDDs/profiles, and (d) simulated dose distribution delivered by a VAMT plan. Based on quantitative comparison with water and Solid Water as reference, FlexyDos3D is found to exhibit excellent water‐equivalence for MV photon, but poor soft tissue equivalent performance for kV photon. The higher HU value of FlexyDos3D measured by kV CT is found to induce underdose to target, which can be eliminated by HU correction. As indicated, from the perspective of radiological tissue equivalence, FlexyDos3D can serve as an acceptable water‐equivalent dosimeter for clinical use for MV radiotherapy x‐ray beams, while the nonwater‐equivalence effect for kV photons requires HU correction for kV CT based dose calculation. If FlexyDos3D is to be used in low energy x‐ray dosimetry, we believe that further corrections on its‐water‐equivalence are needed. The findings of this study provide pertinent reference for further FlexyDos3D characterization.

## CONFLICT OF INTEREST

There are no relevant conflict of interest to disclose.
